# The tripartite motif-containing 24 is a multifunctional player in human cancer

**DOI:** 10.1186/s13578-024-01289-3

**Published:** 2024-08-19

**Authors:** Yuanbing Yao, Sheng Zhou, Yue Yan, Kai Fu, Shuai Xiao

**Affiliations:** 1 Institute of Molecular Precision Medicine and Hunan Key Laboratory of Molecular Precision Medicine, Department of General Surgery, Xiangya Hospital, Central South University, 87# Xiangya Road, Changsha, 410008 Hunan China; 2 Department of Ultrasound, Xiangya Hospital, Central South University, Changsha, Hunan Province China; 3 Yanbian University Medical School, Yanji, Jilin, China; 4 Hunan Key Laboratory of Animal Models for Human Diseases, Central South University, 87# Xiangya Road, Changsha, 410008 Hunan China; 5 Center MOE Key Lab of Rare Pediatric Diseases & Hunan Key Laboratory of Medical Genetics of the School of Life Sciences, Central South University, 87# Xiangya Road, Changsha, 410008 Hunan China; 6 Hunan Key Laboratory of Aging Biology, Xiangya Hospital, Central South University, 87# Xiangya Road, Changsha, 410008 Hunan China; 7 National Clinical Research Center for Geriatric Disorders, 87# Xiangya Road, Changsha, 410008 Hunan China; 8 The First Affiliated Hospital, Department of Gastrointestinal Surgery, Hengyang Medical School, University of South China, 69# Chuanshan Road, Hengyang, 421001 Hunan China

**Keywords:** TRIM24, Transcription regulation, Cell proliferation, Epithelial–mesenchymal transition, Cancer treatment

## Abstract

Tripartite motif-containing 24 (TRIM24), also known as transcriptional intermediary factor 1α (TIF1α), is the founding member of TIF1 family. Recent evidence indicates that aberrant expression of TRIM24, functions as an oncogene, is associated with poor prognosis across various cancer types. TRIM24 exhibits a multifaceted structure comprising an N-terminal TRIM region with a RING domain, B-box type 1 and type 2 domains, and a coiled-coil region, as well as a C-terminal plant-homeodomain (PHD)-bromodomain. The bromodomain serves as a ‘reader’ of epigenetic histone marks, regulating chromatin structure and gene expression by linking associated proteins to acetylated nucleosomal targets, thereby controlling transcription of genes. Notably, bromodomains have emerged as compelling targets for cancer therapeutic development. In addition, TRIM24 plays specialized roles as a signal transduction molecule, orchestrating various cellular signaling cascades in cancer cells. Herein, we review the recent advancements in understanding the functions of TRIM24, and demonstrate the research progress in utilizing TRIM24 as a target for cancer therapy.

## Introduction

**Fig. 1:**
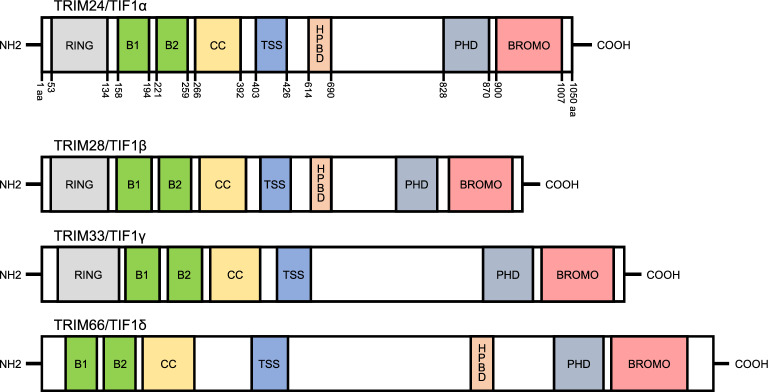
Domain architecture of human TRIM24 (also called TIF1α) and other TIF1 family members, including TIF1β/TRIM28, TIF1γ/TRIM33, TIF1δ/TRIM66. RING: RING domain; B1 and B2: B-box domains; CC: coiled-coil domain; TSS: TIF1 signature sequence; HPBD: heterochromatin protein family binding domain; PHD: plant homeodomain finger; BROMO: bromodomain

TRIM24 is a member of the tripartite motif (TRIM) protein family, which encompasses over 80 proteins characterized by a conserved N-terminal RBBC motif, consisting of a RING domain, one or two B-boxes (B1 and B2), and a coiled-coil (CC) domain [1] (Fig. 1). TRIM proteins play diverse roles in cellular processes, with the RING domain conferring E3 ligase activity within the ubiquitin proteasome system (UPS) [2, 3] while the B-box and CC domains mediate crucial protein–protein interactions [4, 5]. TRIM24 belongs to the C-VI subfamily, along with TRIM28 and TRIM33, characterized by the presence of a plant-homeodomain (PHD) and bromo-PHD fingers read the histone H3 N-terminal domain, mainly the methylation state of H3K4, while bromodomains recognize acetylated lysine [6]. The TIF1 proteins serve as key transcriptional regulators that modulate chromatin state to control the transcription of target genes. Notably, all four TIF1 proteins share a TIF-signature sequence (TSS) essential for transcriptional repression, featuring a central TSS domain characterized by a 25-amino acid sequence rich in tryptophan and phenylalanine [7]. Additionally, TRIM24, TRIM28, and TRIM66 feature heterochromatin protein 1 (HP1) binding domains (HPBD), facilitating interactions with HP1 family proteins through a PxVxL motif [8–12]. This multifaceted classification underscores the involvement of TRIM24 in intricate cellular processes as a versatile protein.

Despite the growing interest in TRIM proteins, their biological role in cancers remains incompletely elucidated. Recent findings highlight that aberrant expression of TRIM24 contributes to cancer development and progression, and this abnormal expression is associated with a poor prognosis in multiple cancers (Table 1). Selective inhibitors of bromodomain have received a significant amount of attention from recently due to their outstanding efficacy demonstrated in multiple cancer models, with several of these inhibitors already advancing to clinical trials recently, which indicates a great potential of TRIM proteins in cancer treatment [13, 14]. This review comprehensively explores the functional properties TRIM24 in cancer cells, providing an updated overview of its current status in cancer research and seeking insights into the profound mechanisms driving tumor progression, as well as potential therapeutic targets associated with TRIM24.

**Table 1:** Alteration and the proposed function of in TRIM24 in human cancer

Cancer type	Alteration	Function	Validated mechanism(s)	References
Acute myeloid leukemia	Increased expression	N/A	N/A	[32]
Bladder cancer	Increased expression	Oncogene	Activating NF-κB and AKT pathways	[30]
Breast Cancer	Increased expression	Oncogene	Binding to chromatin and ER to activate estrogen-dependent genes	[15–17]
Cervical	Increased expression	Oncogene	Activating NF-κB and AKT pathways	[31]
Colorectal cancer	Increased expression	Oncogene	Binding to YAP promoter and activating YAP transcription, activating Wnt/β-catenin pathway	[23–25]
Esophageal squamous cell carcinoma	Decreased expression	N/A	N/A	[37]
Glioma	Increased expression	Oncogene	Activating PI3K/AKT pathway	[33]
Head and neck squamous cell carcinoma	Increased expression	Oncogene	N/A	[26, 27]
Liver	Increased expression	Oncogene	Activating AMPK pathway	[28, 29]
Lung	Increased expression	Oncogene	Regulating cell cycle	[34]
Neuroblastoma	Increased expression	Oncogene	Promoting LSD1/CoREST complex formation	[35]
Ovarian cancer	Increased expression	Oncogene	Activating AKT pathway	[36]
Prostate cancer	Increased expression	Oncogene	Functioning as a transcriptional activator of AR	[18, 19, 22]

## TRIM24 in cancers

### TRIM24 in breast cancer

Aberrant expression of TRIM24, which is increased in human breast cancer, negatively correlates with patient survival and may serve as a new prognostic marker [15]. TRIM24 functions as a chromatin regulator, reading dual histone marks via its PHD-bromodomain. The three-dimensional structure of TRIM24 PHD-bromodomain reveals a single unit that recognizes unmodified H3K4 (H3K4me0) and acetylated H3K23 (H3K23ac) within the same histone tail. TRIM24 binds chromatin and the estrogen receptor to activate estrogen-dependent genes linked to cellular proliferation and tumor development, with its PHD-bromodomain providing a structural rationale for chromatin activation through a noncanonical histone signature [16]. Interestingly, both TRIM24 and H3K23ac levels are higher in human epidermal growth factor receptor 2 (HER2)-positive breast cancer patients, positively correlating with HER2 levels [17]. HER2 overexpression, present in 10%-15% of breast cancer cases, is linked to a worse prognosis but predicts better response to anthracycline, taxane-based chemotherapies, and HER2-targeted therapy. This study highlights the significant role of TRIM24 and H3K23ac in breast cancer, suggesting that TRIM24 small-molecule inhibitors could benefit estrogen receptor- and progesterone receptor-negative or HER2-positive patients [17].

### TRIM24 in prostate cancer

TRIM24 expression was reported to be increased in prostate cancer tissues [18, 19]. TRIM24 serves as an independent prognostic biomarker for prostate cancer, validated across two large independent cohorts [19]. Genome sequencing studies have identified recurring founder mutations within the substrate-binding cleft of speckle-type POZ protein (SPOP), a cullin-RING ubiquitin ligase adaptor, in about 10% of primary prostate cancers [20]. These mutations impair the ability of SPOP proteins to facilitate ubiquitylation and proteasomal degradation of androgen receptor (AR) and its co-activator NCOA3, leading to heightened AR signaling [21]. Consequently, enhanced AR signaling is a distinguishing characteristic of SPOP mutant tumors [22]. TRIM24 plays a crucial role in promoting cell proliferation in SPOP-mutant prostate cancer cells, particularly under conditions of low androgen availability. Mutant SPOP enhances TRIM24 stability, which in turn drives prostate cancer cell growth [22]. TRIM24 not only fosters prostate cancer growth but also sensitizes cells to low androgen levels by binding to gene promoters and activating pathways involved in both cell proliferation and AR signaling. This interaction between AR and TRIM24 directly influences the expression of genes associated with castration-resistant prostate cancer (CRPC) and predicts recurrence in primary tumors. Specifically, bromodomain and its LxxLL motif, crucial for AR interaction, are essential for sustaining CRPC cell proliferation [22]. This dependency on TRIM24 highlights its potential as a therapeutic target, particularly in CRPC where targeting the chromatin-binding capability of TRIM24 could offer new treatment strategies in the future.

### TRIM24 in colorectal cancer

TRIM24 is upregulated in colorectal cancer and negatively correlates with patient survival, with higher TRIM24 expression associated with shorter survival times [23–25]. TRIM24 expression is an independent prognostic factor and may play an important role in colorectal carcinogenesis, serving as a potential prognostic marker for human colorectal cancer [24]. The lncRNA differentiation antagonizing non-protein coding RNA (DANCR)/KAT6A complex recruits TRIM24 by binding to H3K23, leading to its association with the Yes-associated protein 1 (YAP) promoter. This interaction activates YAP transcription, which in turn enhances the proliferation of colorectal cancer cells [23]. TRIM24 significantly influences proliferation, migration, invasion, and stem-like characteristics in colorectal cancer cells. While its expression is linked to the activation of Wnt/β-catenin signaling, the precise mechanism remains to be elucidated [25].

### TRIM24 in head and neck squamous cell carcinoma

TRIM24 is often overexpressed in tumor tissues from patients with head and neck squamous cell carcinoma (HNSCC) [26, 27]. This up-regulation is significantly linked to poorer overall survival in patients with locally advanced HNSCC, establishing TRIM24 as an independent prognostic indicator. Additionally, TRIM24 promotes cell growth and inhibits apoptosis in HNSCC cells in vitro [26]. Differences in TRIM24 expression were observed across various sites of origin in HNSCC primary tumors [27]. No significant difference was found between oral squamous cell carcinomas and hypopharyngeal HNSCC tumors, leading to their grouping as pharyngeal primary tumors. Pharyngeal HNSCC exhibited a significantly lower proportion of TRIM24-positive tumors compared to laryngeal HNSCC and tumors of the oral cavity. Notably, oral cavity HNSCCs had the highest proportion of TRIM24-positive tumors compared to both laryngeal and pharyngeal HNSCC. Of note, TRIM24 levels are elevated in HNSCC primary tumors that exhibit local recurrences [27].

### TRIM24 in hepatocellular carcinoma

In human hepatocellular carcinoma (HCC) tissues, increased expression of TRIM24 protein is observed [28], which is associated with poor differentiation, elevated α-fetoprotein levels, higher rates of intrahepatic metastasis and recurrence, and shorter tumor-free survival times [29]. Additionally, depletion of TRIM24 in HCC cells induces apoptosis, blocks the cell cycle, and inhibits the process of epithelial-mesenchymal transition (EMT) [29]. TRIM24 enhances cell proliferation and migration in human HCC in vitro and accelerates tumor progression in vivo, primarily through AMP-activated protein kinase (AMPK) signaling [28]. Interestingly, elevated TRIM24 levels in HCC samples correlate with higher tumor grade and reduced AMPK expression [28]. These findings highlight the critical role of TRIM24 in HCC progression and suggest potential new therapeutic targets for treating human HCC.

### TRIM24 in other cancers

TRIM24 is overexpressed in bladder cancer and cervical cancer tissues, which drives the proliferation and invasion of cancer cells by regulating NF-κB and AKT signaling pathways [30, 31]. TRIM24 is significantly overexpressed in myelodysplastic syndrome-related acute myeloid leukemia and may play a role in myeloid differentiation by being distinctly regulated in hematopoietic lineages [32]. TRIM24 is elevated in gliomas and positively correlates with tumor malignancy [33]. It promotes tumor growth and enhances chemotherapy resistance through the PI3K/AKT signaling pathway. At the same time, a significant increase in the expression level of TRIM24 was also observed in non-small cell lung cancer (NSCLC), neuroblastoma and ovarian cancer [34–36]. Notably, TRIM24 mRNA and protein levels are reduced in esophageal squamous cell carcinoma tissues, with TRIM24 protein level being considered as an independent favorable prognostic factor for overall survival in esophageal squamous cell carcinoma patients [37].

## Genetic knockout mouse model of TRIM24

To explore the physiological function of TRIM24 in vivo, TRIM24-deficient mice were generated by cre-mediated excision of exon 4 of TRIM24 (TRIM24^dlE4/dlE4^) [38]. Intercrosses of TRIM24^±^ heterozygous mice produced viable offspring at Mendelian ratios, with homozygous mutants displaying no major organ abnormalities or fertility issues up to 2 months postpartum, suggesting that TRIM24 is not essential for development, growth or reproduction in mice. In addition, a large cohort of Trim24-deficient mice were monitored long-term. No changes were observed in major organs, but 80% of male and 69% of female TRIM24 knockout mice developed hepatic tumors between 9 and 29 months, compared to only 4% of age-matched control mice. TRIM24 was considered as a hepatic tumor suppressor, which negatively regulates the interferon (IFN)/signal transducer and activator of transcription (STAT) signaling pathway through retinoic acid receptor α inhibition [38, 39]. Further evidence showed that the TRIM24^dlE4/dlE4^ mouse is not null for TRIM24 in the liver, retaining normal levels of TRIM24 RNA lacking exon 4 [40]. Genetic excision of TRIM24 in conditional knockout mice (TRIM24^dlE1/dlE1^ and TRIM24^hep/hep^) revealed a novel role in hepatic homeostasis, influencing epigenetic regulation of oxidation/reduction, lipid, steroid, and fatty acid metabolism, as well as unfolded protein response and endoplasmic reticulum-stress pathways. The phenotypes were accompanied by inflammation, fibrosis, and progression to HCC without dietary fat manipulation or chemical induction [41]. Interestingly, HCC occurs in the mice without progressing from non-alcoholic fatty liver disease, due to activation of retinoid-dependent enhancers in endogenous murine VL30-retroviral transposons [40]. Somatic hepatocyte-specific knockout of TRIM24, TRIM28, or TRIM33 promotes HCC in mice, with HCC formation upon TRIM24 depletion being strongly potentiated by the additional loss of TRIM33 [42].

While TRIM24 is upregulated in various tumors, including HCC, where it is thought to promote tumorigenesis, its characterization as a hepatic tumor suppressor in genetic knockout mouse models lacks a clear explanation. This divergence may be due to the crucial role of TRIM24 in maintaining hepatic homeostasis, and its over- or under-expression could disrupt balance and contribute to HCC.

## Role of TRIM24 in transcription regulation

### Epigenetic function of TRIM24

TRIM24, first identified as TIF1α, is a critical factor for transcription regulation [38, 43]. Histone post-translational modifications rely on effector proteins, or histone readers, to faithfully interpret their combinations [44, 45]. TRIM24 exhibits multifaceted roles in chromatin regulation, with its PHD-bromodomain serving as a reader capable of recognizing H3K4me0 and H3K23ac within a single histone tail [16]. Genome-wide examination of chromatin interactions reveals that TRIM24 and estrogen receptor alpha exhibit estrogen-dependent binding at specific sites that paradoxically demonstrate estrogen-induced reduction in H3K4me2 levels alongside an augmentation of histone acetylation [16]. Notably, TRIM24 acts as a synergistic activator of the estrogen receptor, promoting breast cancer cell proliferation and contributing to the occurrence of breast cancer.

Lysine acetyltransferase 6A (KAT6A), with its acetyltransferase activity, regulates gene transcription by acetylating histones and interacting with transcriptional factors [46, 47]. KAT6A-induced H3K23 acetylation enhances TRIM24 association with chromatin, leading to the upregulation of phosphatidylinositol-4,5-bisphosphate 3-kinase catalytic subunit alpha (PIK3CA) transcription and activation of the phosphatidylinositide 3-kinase (PI3K)/protein kinase B (PKB, also known as AKT) signaling pathway in glioma cells [48]. The KAT6A-mediated PI3K/AKT signaling is dependent on its acetyltransferase activity and the H3K23/TRIM24 complex. Elevated expression of KAT6A was observed in HCC tissues and cell lines, demonstrating a significant correlation with aggressive prognostic features and reduced survival. Mechanistically, KAT6A was found to promote H3K23ac, thereby enhancing the association between TRIM24 and H3K23ac. Consequently, TRIM24 assumed a role as a transcriptional activator, stimulating the transcription and expression of SRY-box transcription factor 2 (SOX2) and thereby contributing to HCC tumorigenesis [49]. It’s also reported that TRIM24 activated the expression of SOX2, thereby governing the stemness and invasion of glioblastoma both in vitro and in vivo [50]. Another research provided evidence that TRIM24 interaction with H3K23ac, mediated by the DANCR/KAT6A complex, enhances oncogenic processes associated with the YAP signaling pathway in colorectal cancer [23]. Additionally, KAT6A acetylates mothers against decapentaplegic homolog 3 (SMAD3) at K20 and K117, promoting its association with TRIM24 [51]. TRIM24 interacts with SMAD3, leading to the dissociation of SMAD3 from the tumor suppressor TRIM33. Then, TRIM24-SMAD3 complex is recruited to chromatin, which enhances SMAD3 activation and immune response-related cytokine expression, ultimately promotes breast cancer stemness and enhances metastasis in triple-negative breast cancer.

### Protein–protein interaction partners of TRIM24

The cullin-RING ubiquitin ligase adaptor SPOP with PC-specific mutations is reported to be deficient in mediating ubiquitylation and proteasomal degradation of AR and its co-activator, promoting AR signaling [21, 52]. Thus, enhanced AR signaling is recognized as a key characteristic of SPOP mutant tumors [53]. TRIM24, identified as a potential downstream effector protein of SPOP mutations, exhibited reduced ubiquitylation and increased protein levels in the presence of SPOP mutations [54]. A subsequent investigation revealed that TRIM28 interacts with TRIM24, preventing SPOP-mediated ubiquitination of TRIM24 [55]. This interaction results in TRIM24 accumulation and AR signaling activation, thereby promoting the tumorigenesis of prostate cancer. Further research showed that TRIM24 functions as a coactivator of the AR, playing a pivotal role in disease progression by acting as an oncogenic transcriptional activator in prostate cancer cells [22]. Particularly in CRPC settings, TRIM24 collaborates with AR-dependent gene expression to enhance cancer progression. Prostate-specific membrane antigen (PSMA) is a protein specifically expressed on prostate epithelial cells and is notably overexpressed in nearly all prostate cancers [56]. A study assessed the effectiveness of a human monoclonal PSMA antibody (PSMAb)-based platform for targeted delivery of TRIM24 siRNA and its therapeutic impact in CRPC [57]. The PSMAb-mediated TRIM24 siRNA delivery platform demonstrated significant inhibition of cell proliferation, colony formation, and invasion in PSMA-positive CRPC in vitro, and suppression of tumor growth in a xenograft model. Additionally, the identification of bromodomain-containing 7 (BRD7), a negative regulator of cell proliferation and growth, represses the AR transactivation activity induced by TRIM24 and contributing to the modulation of prostate cancer pathogenesis [58]. TRIM24 emerges as an oncogenic transcriptional co-activator in epidermal growth factor receptor (EGFR)-driven glioblastoma [59]. EGFR signaling promotes histone H3 lysine 23 acetylation and association with TRIM24, which functions as a co-activator to recruit signal transducer and activator of transcription 3 (STAT3). This interaction results in stabilized STAT3-chromatin interactions and subsequent activation of STAT3 downstream signaling, thereby intensifying the oncogenic activity of the EGFR/STAT3 pathway in glioblastoma.

Tumor protein 53 (p53), a pivotal tumor suppressor, serves as a master regulator governing genomic stability, cell cycle progression, DNA repair, senescence, and apoptosis [60]. The functions of p53 are often compromised in tumorigenesis, primarily due to somatic mutations, prevalent in over 50% of human cancers. These mutant variants have been demonstrated to acquire oncogenic functions in addition to the loss of tumor-suppressive function of p53, contributing to the development of an aggressive malignant phenotype. In embryonic stem cells, TRIM24 functions as a ubiquitin-protein ligase, promoting p53 degradation to counteract differentiation or induce a conformational shift in p53, thereby preventing cellular transformation [61, 62]. The accumulation of phosphorylated p53 and its activation of genes associated with various DNA repair pathways contribute to its direct role in promoting DNA repair during the DNA damage response (DDR) [63]. TRIM24 functions as a RING-domain E3 ligases targeting p53 for degradation [64]. Epigenetic characteristics of closed chromatin, such as DNA methylation, do not influence p53 binding across the genome. A recent study shows that the local chromatin state is influenced by TRIM24, which binds both p53 and H3K4me0, preferentially localizing to closed chromatin sites. This TRIM24-dependent process affects ability of p53 to open chromatin and activate genes, bridging p53 activity to the local chromatin state and impacting cell viability and gene expression in response to stress [65]. The cyclic GMP-AMP synthase (cGAS)/stimulator of interferon response CGAMP interactor 1 (STING) cytosolic DNA-sensing pathway plays a pivotal role as a key mediator in the innate immune response [66]. Under homeostatic conditions, DNA exonucleases, particularly the 3’-5’ cytosolic exonuclease three prime repair exonuclease 1 (TREX1), play a critical role in degrading cytoplasmic DNA, preventing unwarranted activation of the cGAS/STING pathway [67]. Recent studies have shown that p53 significantly contributes to the degradation of TREX1, influencing the accumulation of cytosolic dsDNA [68]. This regulatory process is mediated by TRIM24, the ubiquitin ligase identified as a transcriptional target of p53. Thus, p53-TRIM24 axis controls the cGAS/STING pathway for tumor suppression, highlighting the intricate regulatory mechanisms orchestrated by these molecular players in maintaining cellular homeostasis and suppressing tumorigenesis.

### Transcriptional targets of TRIM24

A hallmark of cancer is replicative immortality, commonly driven by the reactivation of telomerase reverse transcriptase (TERT or hTERT in humans), the catalytic subunit of telomerase, which is responsible for telomere maintenance [69, 70]. TRIM24 and TRIM28 have emerged as key regulators of hTERT expression in bladder cancer [71]. The recruitment of TRIM28 to the mutant promoter initiates an interaction with TRIM24, leading to the inhibition of TRIM28 function. Intriguingly, the phosphorylation of TRIM28 by mTORC1 plays a pivotal role by disengaging it from the inhibitory interaction with TRIM24, subsequently triggering the induction of hTERT transcription. This complex regulatory interplay between TRIM28 and TRIM24 provides valuable insights into the mechanisms governing hTERT expression in bladder cancer. The oncogenic role of the plasmacytoma variant translocation 1 gene (*PVT1*), a lncRNA located at 8q24.21, has been established in various cancers [72]. In glioma cells, PVT1 recruits COPS5 to deubiquitinate and stabilize TRIM24, leading to the activation of STAT3 signaling and the induction of malignant biological behaviors [73]. TRIM24 negatively regulates STAT1 in HNSCC, contributing to immunosuppression in cancer cells while enhancing T cell antitumor immunity in the tumor microenvironment [74], whereas, the precise mechanism remains elusive. A systematic investigation demonstrates that TRIM24 associates with pluripotency transcription factors, including OCT-3/4, SOX2, and NANOG, on multiple enhancers in stem cells, where it actively suppresses the expression of developmental genes to sustain the pluripotent state [75].

**Fig. 2:**
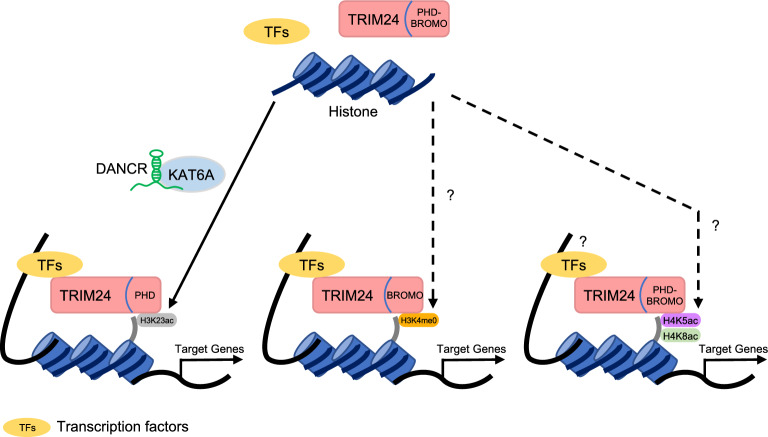
The schematic diagram illustrating how TRIM24 involves in transcription regulation by binding to specific histone sites via distinct domains. H3K23ac: acetylated Histone 3 lysine 23; H3K4me0: unmethylated Histone 3 lysine 4; H4K5ac: acetylated Histone 4 lysine 5; H4K8ac: acetylated Histone 4 lysine 8; KAT6A: lysine acetyltransferase 6A; DANCR: differentiation antagonizing non-protein coding RNA; TFs: Transcription factors

In previous research, the TRIM24 PHD-bromodomain exhibited a preference for recognizing H3K4me0 and H3K23ac (Fig. 2). A recent study focused on different acetylated histone H4 binding partners of TRIM24 to enhance our understanding of its gene-regulatory function [76]. TRIM24 PHD-bromodomain exhibits a preference for binding to H4K5ac, H4K8ac, and H4K5acK8ac in comparison to other acetylated histone H4 ligands utilizing isothermal titration calorimetry in a binding assay with histone peptides. A chromatin immunoprecipitation (ChIP)-seq based analysis unveiled a robust co-localization of the H4K5ac and H4K8ac histone signatures in proximity to transcription start sites for various hub genes or genes targeted by TRIM24 in breast cancer. However, it remains uncertain whether these transcriptional regulation mechanisms still rely on the binding of transcription factors. Furthermore, TRIM24 PHD-bromodomain was found to recognize the H3K4me0 mark via its PHD domain, while simultaneously exhibiting a broad binding spectrum for acetyllysine-containing H4 tails through its bromodomain. These findings demonstrate the diverse histone recognition capabilities of TRIM24 and their significance in the context of breast cancer gene regulation.

## Role of TRIM24 in cancer cell proliferation

**Fig. 3:**
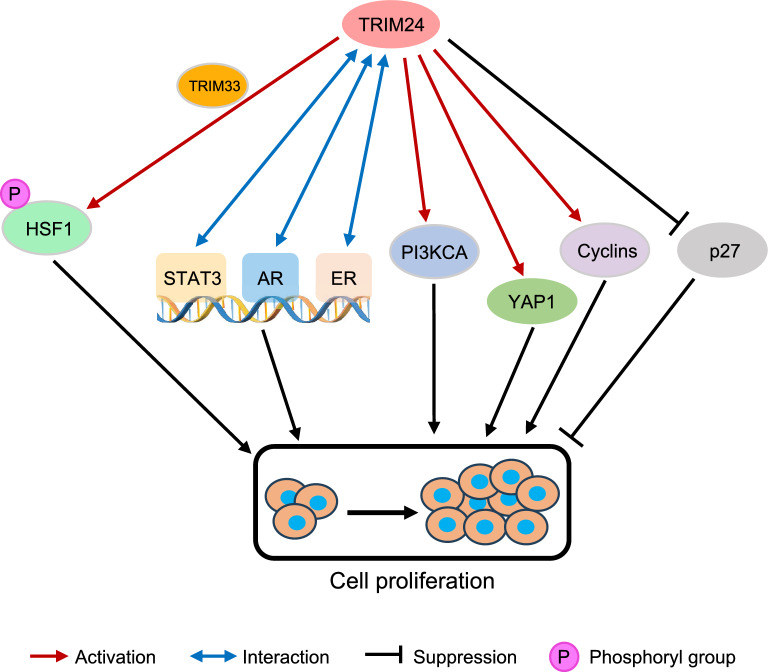
The schematic diagram illustrating the TRIM24-mediated signaling pathway and its effectors in the regulation of cell proliferation. HSF1: heat shock transcription factor 1; STAT3: signal transducer and activator of transcription 3; AR: androgen receptor; ER: estrogen receptor; PIK3CA: phosphatidylinositol-4,5-bisphosphate 3-kinase catalytic subunit alpha; YAP1: Yes1 associated transcriptional regulator; p27: cyclin dependent kinase inhibitor 1B

Cancer is fundamentally characterized by abnormal and uncontrolled cell proliferation, leading to an increase in tumor cell number and burden [77]. Cancer cells exhibit characteristics allowing prolonged survival and abnormal proliferation, with the loss of normal growth control observed not only in early tumor initiation but also during metastasis [78]. Proliferation plays a crucial role in cancer development, involving altered expression or activity of cell cycle-related proteins and the constitutive activation of signaling pathways promoting cell growth [78]. Many studies showed TRIM24 played a crucial role in regulation of proliferation in various cancer types (Fig. 3).

Depletion of TRIM24 in NSCLC and HCC cells impedes cell proliferation by inducing cell cycle arrest at the G1-S transition, while decreasing the percentage of cells in the S and G2 phases [29, 34]. Western blot analysis demonstrates decreased protein levels of Cyclin A, B, D1, E, p-Rb, and elevated p27 expression upon TRIM24 knockdown [34]. These findings collectively indicate that inhibiting TRIM24 expression retards cancer cell growth by disrupting the cell cycle progression at the G1-S transition. TRIM24 was found to bind to the promoter region of Cyclin D1 utilizing ChIP analysis, offering partial clarification of the molecular mechanism underlying its impact on cell proliferation through transcriptional regulation of genes [79], which is consistent with the findings in gastric cancer cells [80].

The recruitment of TRIM24 by DANCR/KAT6A complex, specifically binding to H3K23ac, leads to TRIM24 binding to YAP promoter. This interaction activates YAP transcription, ultimately promoting the proliferation of colorectal cancer cells. The crucial role of TRIM24 in colorectal cancer cell proliferation is facilitated by the DANCR/KAT6A complex, which enhances TRIM24 association with H3K23ac and subsequently recruits YAP to chromatin, contributing to increased cell proliferation [23]. TRIM24 plays a pivotal role in driving the proliferation of castration-resistant prostate cancer cells, aligning with the concept that AR co-activators sustain AR-mediated signaling during low hormone availability. TRIM24, by binding to acetylated histones via its bromodomain, anchors AR to the genome during hormone-starvation conditions [22]. TRIM24 plays a pivotal role in the malignant transformation of normal mammary epithelial cells. Ectopic expression of TRIM24 in immortalized human mammary epithelial cells (HMECs) significantly enhances cellular proliferation and induces malignant transformation, as evidenced by xenograft tumor growth. Moreover, TRIM24 overexpression leads to metabolic reprogramming, activating glycolytic and tricarboxylic acid cycle gene signatures, emphasizing its role in the deregulation of cancer-associated pathways, including glucose metabolism, associated with breast tumorigenesis [81]. Notably, TRIM24 is a co-activator of estrogen receptor-α, facilitating the activation of estrogen-dependent genes linked to cellular proliferation and tumor development [16].

In glioma cells, TRIM24 recruits STAT3 as a transcriptional co-activator, stabilizing STAT3-chromatin interactions, which activates STAT3 downstream signaling and amplifies EGFR-driven cell proliferation. The findings reveal a novel mechanism where H3K23ac/TRIM24 mediates EGFR-induced STAT3 activation, enhancing the oncogenic potential of the EGFR/STAT3 pathway in human cancer [59]. Depletion of TRIM24 in glioma stem cells significantly reduced both p-STAT3 levels and cell proliferation [59], mirroring a similar phenotype observed in nasopharyngeal carcinoma cells [82]. Another study demonstrated that high TRIM24 expression is positively linked to increased glioma malignancy. TRIM24 plays a crucial role in glioma progression by influencing cell proliferation, cell cycle advancement, clone formation, and in vivo tumor development. Mechanistically, TRIM24, through its PHD-bromodomain, interacts with the promoter of PIK3CA, leading to the activation of PIK3CA gene transcription and subsequently activating PI3K/AKT signaling.

The process of transcriptional regulation by RNA polymerase II is intricately linked to alterations in chromatin structure [83]. Activated and promoter-bound heat shock transcription factor 1 (HSF1) engages coactivators comprising chromatin remodeling complexes and histone-modifying enzymes, promoting the assembly of the preinitiation complex that includes RNA polymerase II and general transcription factors [84]. Specifically, HSF1 recruits the TRRAP-TIP60 HAT complex and p300, leading to the establishment of an active chromatin state characterized by histone acetylation and acetylation-dependent H2B mono-ubiquitination catalyzed by TRIM33 and TRIM24 within heat shock protein promoters [85]. The recruitment of these histone-modifying enzymes is prompted by the phosphorylation of HSF1 at serine 419 mediated by polo like kinase 1. Furthermore, this phosphorylation is persistently elevated in cancer cells, promoting their proliferation.

## Role of TRIM24 in epithelial–mesenchymal transition

EMT is a reversible process where epithelial cells transform into mesenchymal cells, acquiring motility and invasive properties [86]. This plastic transition between epithelial and mesenchymal states is crucial in both development and cancer [87]. During the early 1980s, the association between EMT and cancer was documented [88]. This process plays a pivotal role in the progression of benign tumor cells, engaging them with infiltrating and metastasizing properties. In fact, most of tumors undergo EMT during their progression, particularly the cancers originating from epithelia [89]. Activation of EMT leads to the loss of cell polarity and adhesion, transforming tumor epithelial cells into migratory and invasive mesenchymal cells, which enables the cells to adopt a mesenchymal phenotype characterized by enhanced migratory capacity, increased invasiveness, elevated resistance to apoptosis, and a substantial boost in the production of extracellular matrix components [90].

Studies in various cancers, including HCC [29], gastric cancer [91], ovarian cancer [36, 92], renal cell carcinoma [93, 94], colorectal cancer [97], and NSCLC [34], have demonstrated that down-regulation of TRIM24 reduces the migratory and invasive capabilities of cancer cells and influences the expression of EMT-related proteins. miR-339-3p was identified as a direct regulator of KAT6A, influencing EMT of nasopharyngeal carcinoma cells [95]. Furthermore, KAT6A was found to modulate TRIM24 expression by promoting H3K23 acetylation within the TRIM24 promoter region. Notably, TRIM24 is significantly increased in nasopharyngeal carcinoma [82]. The interplay between miR-339-3p, KAT6A, and TRIM24 establishes an axis that plays a crucial role in prohibiting EMT in nasopharyngeal carcinoma cells [95]. In a study using a mouse model, the conditional overexpression of TRIM24 in mouse mammary epithelia leads to the spontaneous development of mammary tumors, with TRIM24 exerting its impact through its function as a histone reader in chromatin association and the disruption of EMT process [96]. Elevated expression of TRIM24 alone is adequate to induce tumors displaying a molecular signature indicative of EMT in mammary tissues.

Despite these findings, the specific mechanism linking TRIM24 to EMT remains unclear. One proposed explanation suggests that TRIM24 influences the EMT process by modulating the Wnt/β-catenin pathway, a crucial factor in EMT and oncogenic transformation leading to cell invasion and metastasis [97]. The Wnt/β-catenin pathway involves nuclear translocation of β-catenin, causing E-cadherin downregulation and EMT induction, while elevated β-catenin levels are linked to invasion and metastasis in various solid tumors [98]. However, the precise mechanism by which TRIM24 impacts the Wnt/β-catenin pathway remains unknown.

## Role of TRIM24 in chemotherapy

### TRIM24 in DNA damage response

Cells encounter DNA damage from various sources, including ultraviolet, ionizing radiation, genotoxic chemicals, and internal processes like metabolic activities and errors in DNA replication [99]. DDR has been recognized as a complex network of cellular pathways that cooperate to sense and repair diverse DNA lesions [100]. While single-strand break is commonly tolerated and efficiently repaired, double-strand break (DSB) poses a greater threat due to their potential for genomic rearrangement and the intricacies involved in their repair [101]. In the face of the devastating DNA damage caused by DSB, cells are equipped with two repair pathways—homologous recombination (HR) initiated by the MRN complex and nonhomologous end-joining (NHEJ) led by the Ku70/80 heterodimer [102, 103]. These components act as central actors in ensuring effective repair and maintaining genomic integrity [104].

**Fig. 4:**
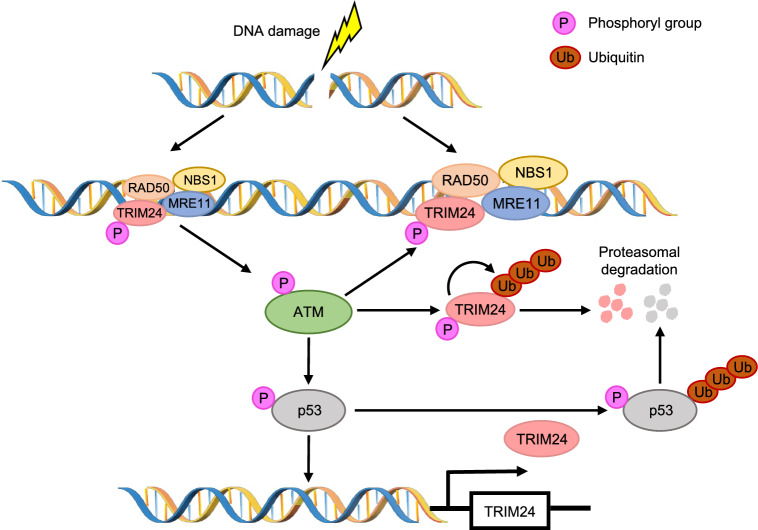
The regulatory mechanisms orchestrated by ATM, p53 and TRIM24 in response to DNA damage. DNA damage triggers the formation of MRN complexes, facilitating ATM activation. Activated ATM phosphorylates downstream substrates, including TRIM24, promoting its enrichment at DNA damage sites and amplifying the recruitment of MRN complex. Then, phosphorylated TRIM24, functioning as an E3 ligase, undergoes self-ubiquitination and degradation. p53 is another downstream substrate of ATM. Phosphorylated p53 induces TRIM24 transcription, and the newly synthesized TRIM24 promotes ubiquitination and degradation of phosphorylated p53, terminating the DNA damage response

A screening of 32 human bromodomain-containing proteins, including all four TIF1 family members, demonstrated that TRIM24, TRIM28, and TRIM33 localized to damage sites in the U2OS osteosarcoma cell line following laser microirradiation [105], while TRIM66 did not exhibit the same behavior. Subsequent investigations indicated that depleting any of the four TIF1 proteins resulted in decreased efficiency of HR repair, with minimal impact on NHEJ [106]. In support of the results of screening, our recent data demonstrated that TRIM24 accumulated in the nuclear regions within seconds following laser microirradiation, which suggests a dynamic response to cellular damage [107]. Furthermore, TRIM24, interacting directly with meiotic recombination 11 homolog A (MRE11) via its PHD-bromodomain and playing an essential role in MRE11-RAD50-NBS1 (MRN) complex recruitment, demonstrates a significant reduction in the repair efficiency of the HR pathway in TRIM24-depleted cells compared to control cells. The dynamic interplay between the MRN complex and ataxia telangiectasia mutated (ATM), the central kinase in response to DSB, is crucial in controlling the response to DSB. Rapid recognition and localization of MRN to DSB facilitate the recruitment and activation of ATM [108]. ATM phosphorylates downstream effectors such as all three MRN complex members, p53, checkpoint kinase 2 (CHK2), breast cancer type 2 susceptibility protein (BRCA2), and H2A histone family, member X (H2A.X), then mediates the subsequent cellular responses [108, 109]. In response to prolonged DNA damage, ATM phosphorylates TRIM24 at serine 768 (Ser768), initiating autoubiquitination and subsequent degradation in cancer cells [110]. Intriguingly, DNA damage induces ATM-mediated phosphorylation of p53, which directly induces the transcription of TRIM24 in response to DNA damage [110]. Newly synthesized TRIM24 interacts with phosphorylated p53 and leads to its degradation, ultimately terminating the DDR. Recent findings further enhance our understanding by revealing that ATM phosphorylates TRIM24 at both Ser768 and serine 808 (Ser808), which facilitates the recruitment of TRIM24 to chromatin, actively involving it in the DSB-induced DDR [107]. These insights indicate the regulatory mechanisms orchestrated by ATM, p53 and TRIM24 in response to DNA damage (Fig. 4).

### TRIM24 in chemoresistance

Chemotherapeutics exert their effects on rapidly dividing cancer cells by inducing DNA damage directly or indirectly [111]. The clinical application of DNA damage-inducing agents remains the frontline choice for many unresectable or metastatic malignancies [112]. These agents that induce DNA damage in cancer cells may lead to cell death, but the emergence of chemotherapy resistance highlights the importance of DNA repair mechanisms in ensuring cell survival [113].

TRIM24, through PI3K/AKT/NF-κB signaling, controls the expression of the DNA repair enzyme O6-methylguanine-DNA methyltransferase (MGMT), contributing to increased resistance to temozolomide, the standard chemotherapeutic agent for glioblastoma [33]. The regulatory role of TRIM24 in the PI3K/AKT/NF-κB pathway influences MGMT expression and, consequently, enhances resistance to alkylating chemotherapy by reducing the cytotoxic effects induced by chemotherapy. In gastric cancer cells, overexpression of TRIM24 alters cell survival in response to 5-Fluorouracil (5-FU) treatment, indicating a potential role in chemoresistance. This effect may be mediated through the activation of the AKT pathway, as evidenced by increased AKT phosphorylation. Consistently, TRIM24-induced chemoresistance to 5-FU can be reversed by the AKT inhibitor LY294002, suggesting a regulatory relationship between TRIM24 and AKT signaling, resulting in chemotherapy in gastric cancer cells [80]. Intriguingly, DNA damage induced by chemotherapeutics triggers the phosphorylation of p53, subsequently promoting the transcription of TRIM24 in response to DNA damage [110]. TRIM24 plays a crucial role in mediating the recruitment of the MRN complex to chromatin in response to DSB. Mechanistically, ATM phosphorylates TRIM24 at Ser768 and Ser808, facilitating its recruitment to DSB sites and promoting the accumulation of MRN components on chromatin. Notably, TRIM24 determines the sensitivity of human hepatocellular carcinoma to etoposide-induced DSB at both the cellular level and in a xenograft tumor model [107]. Circ_RNF13 and DEAD/H (Asp-Glu-Ala-Asp/His) box polypeptide 27 (DDX27) exhibit upregulation in colorectal cancer patient samples and cells. The regulatory network involves circ_RNF13 orchestrating DDX27 expression through TRIM24-mediated transcriptional regulation [114]. Circ_RNF13 contributes to the stabilization of TRIM24 by suppressing F-box and WD repeat domain containing 7 (FBXW7)-mediated TRIM24 degradation, thereby enhancing chemosensitivity in colorectal cancer.

## Prospects of TRIM24 as potential targets in cancer treatment

Bromodomains, specialized protein interaction modules reading acetylated lysine residues, have showed the potential for designing inhibitors that modulate gene transcription and chromatin structure in cancer treatment [115, 116]. With the development of selective chemical probes and clinical inhibitors, Bromodomains, particularly bromodomain and extra-terminal domain (BET), BET inhibitors are emerging as innovative targeted medications in the investigational phase for the therapy of malignant tumors [13, 14]. The TIF1 family proteins, including (TRIM24, TRIM28, TRIM33, and TRIM66), belong to BET subfamily, which possess a dual C-terminal PHD- bromodomain, making them intriguing targets for investigating epigenetic regulation through bromodomain inhibition. The three-dimensional structure of TRIM24 PHD-bromodomain reveals its cooperative recognition of unmodified H3K4 and acetylated H3K23 [16]. In addition, aberrant expression of TRIM24 has been associated with poor prognosis across multiple cancer types as we reviewed above, making this bromodomain-containing protein an attractive target for oncology research.

Members of the bromodomain-containing protein family, TRIM24 and bromodomain and PHD finger containing 1 (BRPF1), are pivotal in epigenetic gene expression regulation, with TRIM24 showed oncogenic properties in human cancer, while BRPF1 acts as a crucial scaffolding protein in histone acetyltransferase complex assembly. The small molecules modified based on the 1,3-benzimidazolone scaffold, including IACS-9571, were identified as a selective high-affinity dual TRIM24-BRPF1 bromodomain Inhibitor, positioning them as promising candidates for further investigations in the development of targeted therapeutic interventions for cancer [117–119]. Recently, TRIM24/BRPF1-IN-2, a novel derivative of 1-(indolin-1-yl)ethan-1-one, functions as a dual inhibitor targeting TRIM24 and BRPF1 was reported [120]. This compound demonstrates substantial efficacy in inhibiting cancer cell growth both in vitro and in a mouse xenograft model, while exhibiting negligible toxicity. In a sequential process involving in-house library screening, structure-based optimization, and molecular docking studies, two compounds based on N-benzyl-3,6-dimethylbenzo[d]isoxazole-5-amines were unveiled as innovative inhibitors of the TRIM24 bromodomain [121]. These compounds demonstrated the ability to inhibit the growth of multiple cancer cells, indicating their potential anti-cancer activities.

Targeted protein degradation, directing proteins to the cells’ own degradation machinery of the cells known as UPS, has emerged as a novel therapeutic strategy in drug development, showing promise for the discovery of novel therapeutics over the last decade [122]. This approach can be implemented through the use of bifunctional heterodimeric ligands, known as proteolysis targeting chimeras (PROTACs), utilizing the UPS and exploits the substrate specificity of E3 ligases to induce the degradation of specific disease-related target proteins, all without disrupting the broader cellular proteome [123]. A breakthrough study introduces the TRIM24-targeting PROTAC (dTRIM24) created by conjugating a TRIM24 inhibitor IACS-9571 with a VHL ligand [124]. This PROTAC effectively and selectively degraded TRIM24, producing an anti-proliferative phenotype in acute myeloid leukemia cells [124]. Recent preclinical studies have shown the significant potential of dTRIM24 in cancer treatment. Conditional TRIM24 overexpression in mouse mammary epithelia leads to the spontaneous development of ER, PR, and HER2-negative mammary carcinosarcomas. Treatment with dTRIM24 significantly reduced cell viability in triple-negative breast cancer PDX tumorspheres compared to the negative control [96]. dTRIM24 inhibited cell propagation and invasion in patient-derived glioblastoma stem cells, partially through suppressing the TRIM24-SOX2 axis [125]. The dTRIM24 has demonstrated the ability to selectively and efficiently degrade TRIM24 and cellular viability of various tumors in vitro, exerting a pronounced impact on genome-wide transcription at TRIM24 target genes, showcasing its potential as a powerful tool in the field of targeted protein degradation [124, 125].

## Other TIF1 family members and cancers

### TRIM28

TIF1 members are aberrantly expressed in multiple cancer types [126, 127]. Like TRIM24, TRIM28 is typically considered as an oncogene, which is expressed higher in tumor tissue when compared to adjacent healthy tissue in various cancers [128–130]. TRIM24 is known to possess E3 ligase activity, though its substrates in cancer cells remain largely unidentified, with p53 being the only confirmed substrate. TRIM24 directly binds to p53, promoting its polyubiquitination and degradation [64, 110]. Similarly, TRIM28 has E3 ligase activity through its RING finger domain, but it functions differently from TRIM24. TRIM28 interacts with MDM2, recruiting cofactors like histone deacetylases to form the p53-HDAC1 complex and inhibit p53 acetylation [131]. Since p53 acetylation and ubiquitination are mutually exclusive, MDM2 recruits TRIM28 to facilitate p53 deacetylation and subsequent ubiquitination. Acting as an E3 ubiquitin ligase, TRIM28 enhances p53 ubiquitination in an MDM2-dependent manner [131, 132]. TRIM28 also promotes chemokine-driven myeloid-derived suppressor cell recruitment in the tumor microenvironment by interacting with RIPK1 (receptor interacting serine/threonine kinase 1) and facilitating its K63-linked polyubiquitination, thereby activating the NF-κB signaling pathway. Besides its E3 ubiquitin ligase activity, TRIM28 also functions as an E3 SUMO ligase, specifically conjugating SUMO2 (but not SUMO1) to PCNA (proliferating cell nuclear antigen) to prevent DNA damage, suggesting a role in DNA damage-induced chemotherapeutic drug resistance [133].

DNA damage induces ATM to phosphorylate TRIM24, leading to p53 degradation and MRN complex recruitment, which promotes DNA repair. Intriguingly, ATM phosphorylates TRIM28 on Ser824 within the C-terminus and Ser473 near the HP1 binding domain early in the DNA damage response [134, 135]. Phosphorylation of Ser824 disrupts NuRD and SETDB1 (SET domain bifurcated histone lysine methyltransferase 1) recruitment, halting heterochromatin compaction, while Ser473 phosphorylation impairs the binding of TRIM28 to HP1 proteins and its transcriptional repression of KRAB-ZNFs target genes. The phosphorylation of TRIM28 results in chromatin relaxation, enhancing DNA repair machinery access (including BRCA1 and 53BP1) to the damage site, thereby promoting tumor progression [136, 137].

Despite studies suggesting the involvement of TRIM24 in regulating EMT in cancer cells, its specific mechanism remains unclear. However, the molecular mechanism of TRIM28 in EMT regulation has been elucidated in several publications, potentially inspiring future research into the role of TRIM24 in EMT. A novel master regulator of EMT, a ternary protein-DNA complex composed of TRIM28, CArG box–binding factor-A (CBF-A), and the fibroblast transcription site-1 (FTS-1) element, induces the expression of fibroblast-specific protein 1 (FSP1), a EMT proximal activator [138]. FTS-1 sites in promoter regions of multiple EMT-related genes are recognized and bound by CBF-A and TRIM28, regulating a wide range of EMT-responsive genes [138]. In NSCLC cells, TRIM28 deficiency blocks transforming growth factor beta (TGF-β)-induced EMT, reducing tumor cell migration and invasion by regulating histone acetylation and methylation on E-cadherin and N-cadherin promoter regions [139]. Additionally, in breast cancer cells, TRIM28 enhances EMT and promotes metastasis by stabilizing TWIST1 [140].

TRIM28 interacts with RLIM, which interacts with MDM2 to promote its degradation, to further enhance its ubiquitination, thereby ensuring low levels of p53 expression and promoting tumor cell proliferation and survival of lung cancer cells [141] Under stress conditions, the absence of TRIM24 resulted in a rapid decrease in numbers of live cells, whereas TRIM24-expressing cells persisted for twice as long before reaching similar levels. Under p53-uninduced conditions, loss of TRIM24 slightly decreased proliferation. Furthermore, genetic deletion of p53 in the TRIM24 degron line demonstrated that the effect on viability after TRIM24 loss requires p53 to be present in cells [65]. However, whether the process of TRIM24 regulating cell proliferation through p53 is dependent on MDM2 still requires further study. Of note, while TRIM28 is widely studied for its promoting effect on cancer cell proliferation [142, 143], it can also exert an anti-proliferative role in early lung cancer by inhibiting the transcriptional activity of the E2F family [144].

## TRIM33

TRIM33, unlike other TIF1 family members, has predominantly been identified as a tumor suppressor [145–147]. Reduced expression of TRIM33 in advanced HCC is associated with shorter overall survival and higher recurrence rates compared to patients with higher TRIM33 expression [145]. TRIM33 monoubiquitinates SMAD4, inhibiting the formation of SMAD transcriptional complexes, and thereby suppresses TGF-β/SMAD signaling which contributes to invasion and metastasis in HCC cells [145]. TRIM33 inactivation leads to chromosomal defects due to weakened spindle assembly and post-mitotic checkpoints, loss of contact growth inhibition, and increased anchorage-independent growth. Clinically, reduced TRIM33 expression in tumors correlates with higher genomic rearrangements, indicating its role in promoting chromosomal stability and tumor suppression [146]. TRIM33, functioning as an E3 ubiquitin ligase, interacts with and ubiquitinates nuclear β-catenin, thereby reducing its abundance. This degradation of nuclear β-catenin is essential for the role of TRIM33 in suppressing tumor cell proliferation and brain tumor development through Wnt/β-catenin signaling [147]. TRIM33 is identified as a downstream target of SOX2 in NSCLC cells, where SOX2 represses TIF1γ transcription, impairing its function and playing a crucial role in TGF-β-induced EMT and cell invasion [148]. Another study demonstrated that TRIM33 inhibits EMT and metastasis in lung adenocarcinoma by restraining TAF15/TBP complex-dependent overactivation of transcription initiation [149]. Of note, somatic hepatocyte-specific inactivation of TRIM24, TRIM28, or TRIM33 promotes HCC in mice, with HCC formation from TRIM24 inactivation being strongly potentiated by further loss of TRIM33 [42].

Contrastingly, some studies have also indicated that TRIM33 plays an oncogenic role in cancers. TRIM33 is identified as a crucial factor in B cell neoplasms, performing its essential function by associating with a single cis element and preventing apoptosis in B lymphoblastic leukemia by interfering with enhancer-mediated Bim activation [150]. An AR-TRIM33 coregulatory gene signature, which is overexpressed in prostate cancer, essential for disease progression, and predictive of recurrence-free survival, was identified. In addition, TRIM33 significantly enhances AR transcriptional activity by preventing Skp2-mediated degradation of AR, thereby promoting prostate cancer growth [151].

Recent research revealed that TRIM33 targets the transcription factor E2F4 for degradation, restricting its interaction with chromatin and the Recql DNA helicase. Deletion of TRIM33 results in constant Recql recruitment to chromatin, accelerated replication forks, and compromised checkpoint signaling and DNA repair under replicative stress, leading to DNA damage accumulation and delayed development of MYC-driven tumors [152]. TRIM33 promotes DNA damage repair by interacting with amplified in liver cancer 1 (ALC1) in a poly(ADP-ribose) polymerase (PARP)-dependent manner and is recruited to DNA damage sites in a PARP1- and ALC1-dependent manner, where it regulates cell cycle progression and facilitates the timely removal of ALC1 from damage sites [153]. A subset of multiple myeloma (MM) patients with TRIM33 copy number loss exhibits poor prognosis and increased chromosomal instability [154]. TRIM33 loss leads to dysregulated ubiquitination of ALC1, the key regulator of response to PARP inhibition, resulting in the accumulation of endogenous DNA damage and slower DNA repair kinetics in MM cell lines. TRIM33 loss-induced DNA damage response defects can be therapeutically targeted using the PARP inhibitor Olaparib [154].

## TRIM66

Although the role of TRIM66 in cancer progression is not well understood, some studies suggest it functions as an oncogene. TRIM66 expression was elevated in CRC tissues and cell lines. Knockdown of TRIM66 inhibited cell proliferation, migration, invasion, and EMT in CRC cell lines through the Janus kinase 2 (JAK2)/STAT3 signaling pathway [155]. Interestingly, another study revealed that TRIM66 promotes the malignant progression of prostate carcinoma through the JAK/STAT pathway, highlighting its importance in the proliferation, cell cycle regulation, migration, and invasion of prostate cancer cells [156]. TRIM66 and MMP9 were upregulated in NSCLC, with TRIM66 facilitating malignant progression through modulation of the MMP9-mediated TGF-β/SMAD pathway. Additionally, TRIM66 interacts with MMP9 and regulates its expression [157]. TRIM66 is overexpressed in human glioma, where it plays a crucial role in proliferation, apoptosis, and glucose uptake, potentially by regulating c-MYC/GLUT3 signaling [158]. However, these studies primarily address the phenotypic aspects, leaving the specific molecular mechanisms of TRIM66 in these cancers yet to be explored.

## Conclusions

In conclusion, TRIM24 is a versatile protein with ubiquitous expression, acting as a member of transcriptional intermediary factor family with functions ranging from histone reading to E3 ligase activity. The diverse biochemical activities and aberrant expressions of TRIM24 have been implicated in its significant role in cancer, rendering it an attractive target for cancer therapy. Moreover, the druggable bromodomain of TRIM24 and its potential as a pharmacological target emphasize its significance in future therapeutic strategies, offering new possibilities for disease prevention, diagnosis, and treatment.

## Data Availability

Not applicable.

## Abbreviations

5-FU: 5-Fluorouracil
AMPK: AMP-activated protein kinase
AR: Androgen receptor
ATM: Ataxia telangiectasia mutated
B1: B-box 1
B2: B-box 2
BET: Bromodomain and extra-terminal domain
BRCA2: Breast cancer type 2
BRD7: Bromodomain-containing 7
BRPF1: Bromodomain and PHD finger containing 1
CC: Coiled-coil
cGAS: Cyclic GMP-AMP synthase
ChIP: Chromatin immunoprecipitation
CHK2: Checkpoint kinase 2
CRPC: Castration-resistant prostate cancer
DANCR: Differentiation antagonizing non-protein coding RNA
DDR: DNA damage response
DDX27: DEAD/H (Asp-Glu-Ala-Asp/His) box polypeptide 27
EGFR: Epidermal growth factor receptor
EMT: Epithelial–mesenchymal transition
FBXW7: F-box and WD repeat domain containing 7
H2A.X: H2A histone family, member X
H3K23ac: Acetylated lysine 23 of histone H3
H3K4me0: Unmethylated H3K4
HCC: Hepatocellular carcinoma
HER2: Human epidermal growth factor receptor 2
HMECs: Human mammary epithelial cells
HNSCC: Head and neck squamous cell carcinoma
HP1: Heterochromatin protein 1
HPBD: Heterochromatin protein 1 binding domains
HR: Homologous recombination
HSF1: Heat shock transcription factor 1
JAK: Janus kinase
KAT6A: Lysine acetyltransferase 6A
MGMT: O^6^-methylguanine-DNA methyltransferase
MRE11: Meiotic recombination 11 homolog A
MRN: MRE11-RAD50-NBS1
NHEJ: Nonhomologous end-joining
p53: Tumor protein 53
PARP: Poly (ADP-ribose) polymerase
PHD: Plant-homeodomain
PIK3CA: Phosphatidylinositol-4,5-bisphosphate 3-kinase catalytic subunit alpha
PKB: Protein kinase B (also known as AKT)
PROTACs: Proteolysis targeting chimeras
PSMA: Prostate-specific membrane antigen
PVT1: Plasmacytoma variant translocation 1
SMAD3: Mothers against decapentaplegic homolog 3
SOX2: SRY-box transcription factor 2
SPOP: Speckle-type POZ protein
STAT3: Signal transducer and activator of transcription 3
TERT: Telomerase reverse transcriptase
TGF-β: Transforming growth factor beta
TIF1: Transcriptional intermediary factor 1
TREX1: Three prime repair exonuclease 1
TRIM: Tripartite motif
TRIM24: Tripartite motif-containing 24
TSS: TIF-signature sequence
UPS: Ubiquitin proteasome system
YAP: Yes-associated protein 1
